# Fungal Bioactive Compounds as Emerging Therapeutic Options for Age-Related Neurodegenerative Disorders

**DOI:** 10.3390/ijms26104800

**Published:** 2025-05-16

**Authors:** Valentina Bonetto, Alessandra Ferraresi, Simonetta Sampò, Ciro Isidoro

**Affiliations:** 1 Department of Science and Technologic Innovation, Università del Piemonte Orientale, 15121 Alessandria, Italy; 2 Department of Health Sciences, Università del Piemonte Orientale, Via Paolo Solaroli 17, 28100 Novara, Italy; alessandra.ferraresi@med.uniupo.it; 3 Department for Sustainable Development and Ecological Transition, Università del Piemonte Orientale, 13100 Vercelli, Italy; simonetta.sampo@uniupo.it

**Keywords:** aging, neurodegeneration, autophagy, mushrooms, polysaccharides

## Abstract

Aging is a complex biological process characterized by progressive multiorgan deterioration that compromises the quality of life. Unhealthy aging often associates with cognitive decline and motor-neurological disorders including Alzheimer’s disease, Parkinson’s disease, and Huntington’s disease. Genetic, environmental, and lifestyle factors, which include dietary habits, interact with aging and influence brain health, thus having an impact on the development of neurodegenerative disorders. In this context, fungal-derived bioactive compounds have emerged as promising neuroprotective agents due to their diverse biological properties that include antioxidative, anti-inflammatory, pro-autophagic, and neurotrophic effects. Key fungal metabolites, including polysaccharides, terpenoids, alkaloids, and phenolic compounds have been shown to modulate neuroinflammatory pathways, enhance neuronal survival, stimulate protective autophagy, and promote synaptic plasticity. Still, challenges related to their bioavailability, standardization, and clinical translation remain unresolved. Future deep research will be crucial to unlocking the full therapeutic potential of fungal-derived neuroprotective compounds. This review examines the potential therapeutic role of fungal metabolites, providing a comparative evaluation with a focus on their mechanisms of action in promoting brain health and longevity.

## 1. Introduction

Aging is an inevitable biological process characterized by a gradual decline in physiological functions, leading to an increased susceptibility to various age-related diseases [1]. While the underlying mechanisms of aging are complex, oxidative stress, chronic low-grade inflammation, and cellular senescence are considered key contributors to this process [1,2]. Additionally, dysfunctional autophagy, a process that preserves cellular and molecular integrity through the lysosome-driven degradation and recycling of oxidized and damaged cellular components, has been found to be associated with aging and age-related neuronal deterioration [1].

Among the most concerning consequences of aging is the rising prevalence of neurodegenerative diseases, such as Alzheimer’s (AD) and Parkinson’s (PD), which pose significant challenges for healthcare systems worldwide. Given their complex pathogenesis and the limited efficacy of current treatments, there is a growing interest in innovative therapeutic strategies. In this context, natural bioactive compounds have emerged as a promising opportunity to address these challenges [3].

Bioactive compounds derived from plant sources, including fruits and vegetables, roots, seeds, and edible flowers, have been suggested to exert anti-aging effects [4,5]. Mushrooms have long been used in traditional medicine, and they are increasingly being studied for their wide range of bioactive compounds. These include structurally important polysaccharides such as chitin and β-glucans, which although primarily known for their roles in fungal cell wall integrity also exhibit immunomodulatory and antioxidant effects [6]. In addition to these, fungi produce metabolites (e.g., phenolic compounds, terpenoids, peptides, and sterols) that have demonstrated therapeutic potential against aging and age-related diseases. Numerous studies have supported the antioxidant, anti-inflammatory, pro-autophagic, neuroprotective, and immunomodulatory properties of these compounds, suggesting that mushrooms represent a promising reservoir of natural agents for promoting health during aging [6,7].

Preventing and alleviating the diseases of aging and improving quality of life are two of the ultimate goals of aging research. This review aims to provide an overview of the role of fungal bioactive compounds in neuroprotection as potential therapeutic agents for age-related neurodegenerative diseases.

## 2. Aging Mechanisms and Biomarkers

Aging is a complex and multifactorial biological process characterized by the progressive decline of physiological functions, increased susceptibility to diseases, and reduced regenerative capacity [8]. While chronological aging is inevitable, biological aging varies significantly among individuals and is influenced by genetic, environmental, and lifestyle factors, including dietary habits.

The hallmarks of aging proposed by López-Otín et al. (2013) [9] provide a framework for understanding the biological basis of aging. These hallmarks include genomic instability, telomere attrition, epigenetic alterations, loss of proteostasis, deregulated nutrient sensing, mitochondrial dysfunction, cellular senescence, stem cell exhaustion, and altered intercellular communication (Table 1). Among these, oxidative stress, chronic inflammation, and cellular senescence are particularly relevant in age-related physiological decline [3]. Oxidative stress, characterized by an imbalance between the production of reactive oxygen species and the body’s ability to detoxify them, can lead to damage to lipids, proteins, and DNA, contributing to the aging process [8]. Elevated levels of oxidative stress biomarkers like malondialdehyde and reduced activities of antioxidant enzymes such as superoxide dismutase and glutathione peroxidase can provide insights into the degree of oxidative damage and imbalance in the body [3].

Chronic inflammation prevalent in elderly people, known as “inflammaging”, is driven in the brain by increased microglial activation and elevated levels of pro-inflammatory cytokines like IL-6 and TNF-α [3]. Furthermore, higher levels of expression of pro-senescence proteins like p16 and p21 induce the permanent growth arrest of cells, which can lead to the accumulation of senescent cells and the release of inflammatory factors, supporting the pro-inflammatory state and further exacerbating the aging process [9,10].

**Table 1:** Biological Mechanisms and Biomarkers involved in the aging process.

Biological Mechanism	Biomarker	Reference
Genomic Instability	DNA damage accumulation, γ-H2AX foci, micronuclei formation	[11,12]
Telomere Attrition	Telomere length, TERT expression	[11,13]
Epigenetic Alterations	DNA methylation patterns (Epigenetic clocks), histone modifications	[14,15]
Mitochondrial Dysfunction	Mitochondrial DNA copy number, ATP production, ROS levels	[16,17,18,19]
Cellular Senescence	Senescence-associated β-gal, p16 INK4a, SASP factors	[3,20]
Chronic Inflammation	IL-6, TNF-α, CRP, NF-κB activation	[2,21,22,23]
Loss of Proteostasis	Amyloid-β (Aβ) aggregation, tau tangles, heat shock protein expression	[24,25]
Stem Cell Exhaustion	Decline in hematopoietic and mesenchymal stem cell markers (CD_34_^+^, CD_133_^+^)	[9,12,26]

Taken together, these processes drive the onset and progression of aging-related diseases, including neurodegenerative disorders, cardiovascular diseases, and metabolic syndromes, by disrupting cellular homeostasis and tissue function [9,21,27].

### 2.1. Neurodegenerative Process

The pathogenesis of neurodegeneration in aging is complex, driven by multiple interconnected processes, including oxidative stress, chronic inflammation, defective autophagy, and cellular senescence. Oxidative stress results from an imbalance between reactive oxygen species (ROS) and the body’s antioxidant defenses, leading to cellular damage that accelerates the decline of neural functions [1]. Chronic low-grade inflammation, common in elderly people, plays a pivotal role in neurodegeneration, as persistent inflammatory responses in the brain contribute to neuronal damage and exacerbate the progression of neurodegenerative diseases [21,22]. Dysfunctional autophagy compromises the clearance of mutated protein aggregates and oxidized mitochondria in neurons, thus favoring neurodegeneration [28]. Additionally, cellular senescence, a state of irreversible cell cycle arrest, has been implicated in neurodegenerative processes, as senescent cells release pro-inflammatory cytokines and proteases that contribute to the breakdown of neural tissue [20,21].

These mechanisms are interconnected in such a way that their cumulative effect accelerates neuronal death and functional decline, particularly in regions of the brain that are highly susceptible to age-related damage, such as the hippocampus and the substantia nigra [23].

### 2.2. Neural Stem Cells in Aging

Neural stem cells (NSCs) are self-renewing, multipotent cells essential for central nervous system (CNS) development, giving rise to neurons, astrocytes, and oligodendrocytes. After neurodevelopment, NSCs end proliferation in most CNS regions, either differentiating terminally or entering a quiescent state. However, certain specialized niches in the postnatal and adult brain, such as the subgranular layer of the hippocampal dentate gyrus, the subventricular zone, and hypothalamus continue to support neurogenesis. Within these niches, NSCs cycle between quiescent (qNSC) and activated (aNSC) states, producing neural progenitor cells (NPCs) that differentiate into neurons and glial cells. These newly generated neurons migrate and integrate into preexisting neural circuits, contributing to brain plasticity [29].

**Figure 1:**
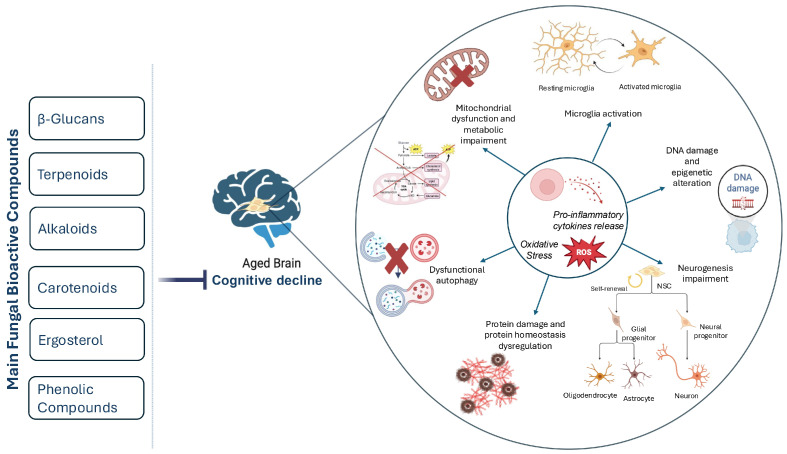
Schematic representation of the key mechanisms of NSC aging and the potential modulatory effects of the main fungal bioactive compounds. The aged brain is characterized by neuroinflammation and oxidative stress. This causes damage to cellular components such as proteins and DNA, leading to alterations in gene expression patterns and disrupted proteostasis. Mitochondrial dynamics change during aging, affecting NCS metabolism. These alterations impair adult neurogenesis and prevent the differentiation of NSCs into neurons and glial cells. Furthermore, aging triggers pro-inflammatory cytokine release and microglial activation, exacerbating neuroinflammation and hampering adult neurogenesis, ultimately affecting cognitive decline. The major classes of fungal bioactive compounds listed can mitigate these pathological mechanisms and support cognitive function.

The declining ability of NSCs to transition from quiescence to an active state is another key mechanism of brain aging. During aging, the proliferation and differentiation potential of neural stem cells decreases, leading to impaired neuronal regeneration and cognitive decline [9]. Over the course of their lives, NSCs accumulate several defects, including a failure to maintain a healthy proteome, metabolic alterations, DNA damage, and epigenetic drift (Figure 1). It is now recognized that, in addition to these intrinsic mechanisms, extrinsic changes in the NSC niche and systemic environment are the primary contributors to NSC aging, and that these mechanisms are not mutually exclusive but rather interrelated and interactive with each other [22]. Structural and molecular changes, including decreased vascular support and the reduced availability of trophic factors, reduce niche support and create a hostile niche for NSCs, impairing their function [12].

Hormonal changes associated with aging also impair NSC function. Declines in systemic factors such as growth hormones and insulin-like growth factor-1 (IGF-1) reduce the regenerative capacity of NSCs. These hormonal alterations affect signaling pathways critical for NSC maintenance and neurogenesis [30]. Indeed, aged NSCs exhibit increased expression of GLUT4, an insulin-dependent glucose transporter, leading to elevated glucose uptake [31]. This higher glucose influx can exacerbate mitochondrial stress, promoting oxidative damage and accelerating NSC aging [30,32].

Mitochondrial dysfunction is another factor associated with cellular aging. Aging disrupts mitochondrial dynamics, favoring excessive fission over fusion, leading to fragmented and inefficient mitochondria [23,32,33]. This imbalance reduces oxidative phosphorylation, forces NSCs to rely more on glycolysis, a less efficient energy pathway, and increases oxidative stress, overwhelming the antioxidant defense system and causing oxidative damage to mitochondrial components [17,18]. Damaged mitochondria remain in NSCs because of dysfunctional mitophagy, the selective degradation of damaged mitochondria via autophagy, associated with increased levels of p53 and p21, which drive NSCs into a quiescent or senescent state [34]. A quiescent state can be also caused by altered gene expression patterns, induced by epigenetic modifications like DNA methylation and histone acetylation [15].

Genomic instability also plays a crucial role. The accumulation of DNA damage over time and telomere shortening compromises NSCs’ survival, function, and proliferative capacity, diminishing their capacity to generate new neurons. This genomic instability is a hallmark of aging and contributes to the functional decline of NSCs [12].

All these conditions, together with chronic low-grade inflammation, as previously reported, negatively impact NSC survival and differentiation, limiting NSC proliferation and neurogenesis. A smaller NSC pool and waning adult neurogenesis bring a higher risk of cognitive impairment and neurodegenerative diseases [2].

### 2.3. Age-Related Neurodegenerative Diseases

The increasing prevalence of age-related neurodegenerative diseases presents a significant challenge to public health systems worldwide. AD and PD represent the most prevalent forms of neurodegenerative disorders, both of which are characterized by progressive neuronal dysfunction and cognitive decline [35]. AD is the most common form of dementia and is characterized by the accumulation of amyloid-beta plaques and neurofibrillary tau tangles, which disrupt synaptic function and lead to the progressive loss of memory and cognitive functions [36]. PD, on the other hand, primarily affects motor skills due to the progressive degeneration of dopaminergic neurons in the substantia nigra, leading to tremors, rigidity, and bradykinesia [37].

Several cellular and molecular mechanisms contribute to age-related neurodegeneration, including mitochondrial dysfunction, oxidative stress, chronic neuroinflammation, and protein aggregation, as previously reported. In AD, mitochondrial dysfunction accelerates β-amyloid (Aβ) toxicity, whereas in PD it contributes to dopaminergic neuron loss in the substantia nigra [19]. Moreover, chronic neuroinflammation, sustained by microglial activation, leads to an overproduction of inflammatory cytokines [21]. This prolonged inflammatory response exacerbates synaptic loss and neuronal death, contributing to the pathology of both AD and PD [36,37,38]. In addition, recent studies have identified Limbic-Predominant Age-Related TDP-43 Encephalopathy (LATE) as a distinct but overlapping neurodegenerative condition that affects the elderly, mimicking AD but involving TDP-43 protein deposits rather than Aβ plaques [39]. LATE predominantly affects memory-related regions such as the hippocampus and is prevalent in approximately one-third of individuals over the age of 85 [25].

Given the multifactorial nature of age-related neurodegenerative diseases, emerging therapeutic strategies focus on targeting fundamental aging mechanisms. These include senolytic therapies to eliminate senescent cells, mitochondrial enhancers, such as NAD+ precursors to improve bioenergetics, and anti-inflammatory approaches, aimed at modulating neuroinflammation [40]. As research progresses, understanding these interconnected aging mechanisms will be essential for developing interventions to preserve cognitive function and mitigate neurodegenerative disease progression in aging populations.

The pathophysiology of these diseases shares several common features, including protein aggregation, mitochondrial dysfunction, impaired autophagy, and neuroinflammation. Despite the advances in understanding the molecular mechanisms underlying these diseases, treatment options remain limited, primarily focused on symptomatic relief rather than halting or reversing disease progression.

### 2.4. Autophagy in Aging and Age-Related Neurodegeneration

**Figure 2:**
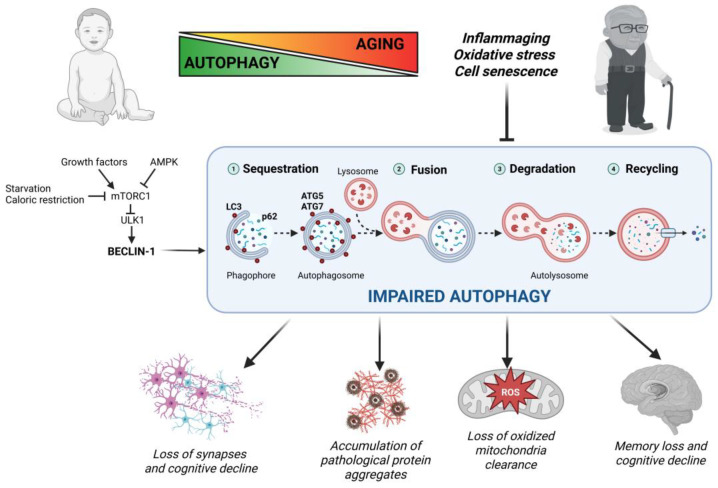
Autophagy impairment accelerates age-related neurodegeneration. Autophagy is regulated by several signaling pathways that converge on mTORC1, which acts as a negative regulator of autophagy. Growth factors promote, while nutrient starvation, caloric restriction, and AMPK activation (e.g., upon energy depletion) suppress mTORC1 activity. Activation of the ULK1 complex (which is inhibited by mTORC1-mediated phosphorylation) promotes the formation of the BECLIN-1-Vps34 pro-autophagic complex. Autophagy starts with the nucleation and elongation of omega-shaped membranes (the phagophore), which will close entrapping the p62-bound cargo to form a double-membrane named autophagosome and decorated with LC3. This process involves, among others, ATG5 and ATG7 proteins. The autophagosome fuses with many lysosomes to form the autolysosome, wherein the cargo is degraded by acidic hydrolases and the subunits are recycled for the synthesis of new cellular components. Aging is characterized by a progressive decline in the efficiency of autophagy clearance, due to excessive inflammation, increased oxidative stress, and cell senescence. Impaired autophagy leads to the accumulation of toxic aggregates and dysfunctional organelles, which in turn exacerbate cognitive decline and memory loss by negatively affecting neuronal survival and synaptic plasticity. Abbreviations: AMPK, Adenosine Monophosphate-activated Protein Kinase; ATG, Autophagy Related Gene; LC3, Light Chain 3; mTORC1, mammalian Target of Rapamycin Complex 1; ULK1, Unc-51 Like autophagy activating Kinase 1; Vps34, Vacuolar Protein Sorting 34 (aka PI3 kinase class III).

Autophagy refers to a catabolic process where damaged, aged, super-oxidized, or redundant organelles and subcellular components are degraded within the lysosomes and the elementary subunits are recycled for resynthesis. Autophagy is a formidable pathway for quality control and macromolecular turnover that maintains cellular homeostasis and keeps the cell “young and healthy” [28,41]. Three pathways for lysosomal degradation have been described, namely macroautophagy, microautophagy, and chaperon-mediated autophagy, with the former playing a major role in organelle, membranes, and macro-aggregates clearance [42]. Here, we will not delve into details of the molecular mechanisms and regulatory pathways of macroautophagy, which have been comprehensively illustrated elsewhere [28,41]. Briefly, autophagy is fine-tuned by several signaling pathways that integrate intra- and extracellular signals (e.g., availability of growth factors, glucose and amino acids, and energy depletion), which converge on mTOR complex 1 (mTORC1). In the presence of amino acids and growth factors, mTORC1 inhibits autophagy through the phosphorylation of the ULK1 complex, which prevents the formation of BECLIN-1/Vps34 pro-autophagic complex [28]. In contrast, mTORC1 is inhibited, and thus autophagy is activated, when the cell lacks glucose or mitochondrial production of ATP is impaired, in which cases AMPK directly activates ULK1 while in parallel inhibiting mTOR [28]. Once autophagy is activated, the cargo is sequestered via the p62/SQSTM1 (Sequestosome 1) into an LC3-labeled double-membrane autophagosome that will fuse with lysosomes to form the autolysosomes, wherein the material is extensively degraded by the acid hydrolases (Figure 2). As a response to stress, autophagy is induced when the cell experiences organelle dysfunction as a result of oxidative stress, nutrient starvation, or the production of genetically altered proteins [43]. Aging is characterized by a progressive deterioration in cellular functions, and not surprisingly this has been linked to dysfunctional autophagy [44,45]. Supporting this connection, genetic impairment of the autophagy process accelerates aging and favors age-related diseases, while vice versa genetic and pharmacologic stimulation of autophagy promotes longevity and prevents age-related disruption of tissue and systemic homeostasis [46,47,48]. As cells age, autophagosome formation, fusion with lysosomes, lysosomal acidification, and the expression of acid hydrolases decline, leading to the accumulation of toxic macromolecules and dysfunctional organelles [49,50,51,52,53,54,55,56,57,58,59,60]. This is particularly pronounced (and dangerous) in mitotically arrested cells, such as neuronal cells, because the accumulated damaged material cannot be diluted through mitosis [52]. Autophagy is downregulated in aged neuronal cells alongside the reduced expression of autophagy genes such as *BECN1*, *ATG5*, and *ATG7* [47,48,49,50]. Autophagy plays a role in age-associated cognitive decline and loss of memory [57,58,59], as well as in age-related neurodegenerative disorders [28,60]. Perturbation of the autophagy-lysosomal pathway is a pathogenic characteristic of tauopathies and Alzheimer’s disease [60,61]. Huntington’s disease exacerbates with aging as the mutated polyQ huntingtin protein (mHTT) abnormally accumulates over time in striatal neurons. It has been shown that mHTT interferes with autophagosome and autolysosome formation [62], and stimulation of autophagy helps to clear the aggregated mHTT and improves neuronal survival [63]. Impaired autophagic degradation of α-synuclein aggregates and of dysfunctional mitochondria in the dopaminergic neurons of the substantia nigra plays a pivotal role in the pathogenesis of Parkinson’s disease [64,65]. Dysfunctional autophagy has also been implicated in the pathogenesis of motor neuron disease and frontotemporal dementia [28].

## 3. Fungal Bioactive Compound Involved in Neuroprotection

The landscape of fungal bioactive compounds in neurodegenerative diseases is a burgeoning field of study, driven by the urgent need for innovative therapeutic approaches to mitigate aging-related neurodegenerative disorders (Table 2).

The biochemical compositions of mushrooms include a plethora of compounds which have been shown to mitigate oxidative damage and modulate inflammatory pathways. These properties are vital in slowing the progression of neurodegenerative disorders, as oxidative stress and neuroinflammation are well-documented contributors to neuronal degradation and cognitive decline.

**Figure 3:**
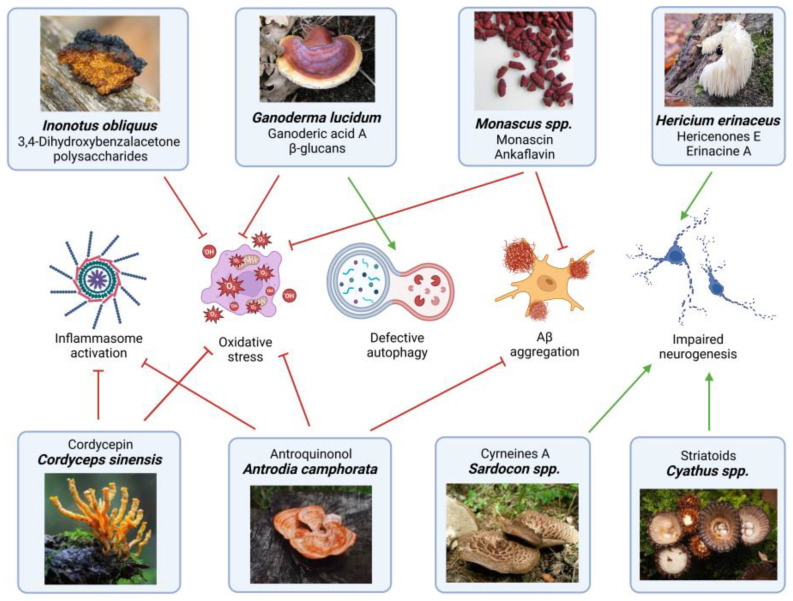
Effects of fungal-derived metabolites in the prevention and/or mitigation of neurodegenerative diseases. Schematic representation of the main biological activities of major bioactive compounds extracted from various fungal species. The illustration highlights their roles in modulating inflammasome activation, oxidative stress, autophagy, protein aggregate accumulation, and neurite outgrowth. Synergistic beneficial effects can be achieved with formulas combining two or more extracts at appropriate concentrations. Green arrows indicate activation/induction, while red arrows indicate inhibition of the respective processes.

Fungal bioactive compounds have emerged as promising neuroprotective agents due to their diverse biological activities, including antioxidant, anti-inflammatory, pro-autophagic, and neurotransmitter-modulating properties, all processes strongly altered in neurodegenerative diseases. Several species belonging to the phylum *Basidiomycota*, such as *Ganoderma lucidum* (Reishi), *Hericium erinaceus* (Lion’s Mane), *Inonotus obliquus* (Chaga), and *Cordyceps sinensis* (Zombie fungus), are known as medicinal mushrooms and are largely employed in Chinese traditional medicine. They contain bioactive metabolites, such as polysaccharides, triterpenoids, ergothioneine, flavonoids, and phenolic compounds, which have been shown to mitigate neurodegeneration and promote cognitive health, making them promising candidates for therapeutic interventions against neurodegenerative diseases [90,91]. Terpenoids, for example, have been found to inhibit glial secretion of pro-inflammatory cytokines, thereby reducing chronic inflammation in the brain [91]. Fungal cell membranes contain high levels of ergosterol, which plays essential roles in cell permeability, fluidity, regulation, and cycle control [88]. The concentration of ergosterol varies by fungal species, developmental stage, and environmental factors, with genera such as *Aspergillus*, *Penicillium*, *Fusarium*, and *Rhizopus* showing levels between 0.4 and 14.3 g/mg dry mass [92,93]. Ergosterol exhibits diverse biological activities, including antioxidant [94,95], anti-inflammatory [96,97], anticancer [98,99], and anti-hypercholesterolemic effects, reducing cardiovascular disease risk [98]. Additionally, ergosterol serves as a precursor to vitamin D2 (ergocalciferol), which exerts neuroprotective effects by modulating cytokine release, cell differentiation, and neurotransmitter signaling, proving beneficial in various neurodegenerative disorders [88]. The fungal cell wall is rich in β-glucans, compounds with biological activity that have been reported to regulate microglial activation, preventing excessive neuroinflammation and protecting neuronal integrity. These polysaccharides exhibit immunomodulatory effects by interacting with pattern recognition receptors on immune cells, leading to the suppression of pro-inflammatory pathways involved in neurodegenerative processes [92]. Moreover, fungal metabolites have recently emerged as autophagy inducers, making them potential candidates for therapeutic applications in age-related diseases, including neurodegenerative disorders [100]. Given their multifaceted neuroprotective effects, fungal bioactive compounds hold great potential as natural therapeutics for preventing or managing age-related neurodegeneration. Future research should focus on clinically validating these compounds and exploring synergistic formulations that enhance their bioavailability and efficacy in human neurodegenerative conditions. In fact, the combination of several compounds, even if absorbed at low concentration, can still produce an overall beneficial effect by acting on different targets and pathways simultaneously (Figure 3). For instance, ethanol extracts of *Ganoderma lucidum* and *Hericium erinaceus* combine anti-inflammatory, pro-autophagic, and pro-neurogenic activities that overall provide neuroprotection. Similarly, extracts from *Inonotus obliquus* and *Antrodia camphorate* could synergistically protect against neurodegenerative disorders thanks to their antioxidant and anti-neuroinflammation properties.

### 3.1. Antioxidant and Anti-Inflammatory Activity

Oxidative stress plays a pivotal role in neuronal aging and neurodegenerative disorders, as excessive production of reactive oxygen species (ROS) leads to mitochondrial dysfunction, lipid peroxidation, and DNA damage, ultimately causing neuronal apoptosis [35]. Fungal bioactive compounds, particularly those derived from edible mushrooms, are rich in antioxidants that play a crucial role in combating oxidative stress [101]. This antioxidant capacity helps in neutralizing free radicals, thereby protecting neuronal integrity and function. *Inonotus obliquus* is a black-brown plant parasitic fungus that belongs to the family *Hymenochaetaceae.* It contains potent antioxidants, including isocoumarins and cyclic diarylheptanoids and flavonoids that significantly enhance antioxidant enzyme activity, including superoxide dismutase (SOD) and catalase (CAT), thereby protecting neuronal cells from oxidative damage [73]. *Ganoderma lucidum*, a woody-inhabiting basidiomycetous fungus belonging to the family *Ganodermataceae*, is known as the “mushroom of immortality” and its polysaccharides and triterpenoids have been largely demonstrated to enhance antioxidant enzyme activity, such as SOD and CAT, thereby mitigating oxidative damage in aging neural tissues [68,90,91].

Furthermore, *Hericium erinaceus* contains erinacines and hericenones, which have been demonstrated to reduce oxidative stress while stimulating nerve growth factor (NGF) synthesis, promoting neurogenesis and neuronal survival [69]. In *G. lucidum*, triterpenoids and polysaccharides act as free radical scavengers, reducing oxidative stress-induced neurotoxicity [90,91]. Additionally, ergothioneine, a naturally occurring antioxidant found in mushrooms, has been found to cross the blood-brain barrier and accumulate in brain regions vulnerable to oxidative stress, where it exerts neuroprotective effects by stabilizing mitochondrial function and reducing protein aggregation, a hallmark of neurodegenerative diseases [102]. Different Ascomycota and Zygomycota such as *Penicillium* spp., *Actinomucor* spp., *Mucor* spp., *Neurospora* spp., *Rhizopus* spp. and *Aspergillus* spp. are well known for L-carnitine (β-hydroxy-γ-N-trimethylaminobutyric acid) production through fermentation processes. L-carnitine is a quaternary ammonium compound that plays a crucial role in energy metabolism, enhancing the transfer of fatty acids and making them accessible for mitochondrial β-oxidation [103]. However, it also acts as an antioxidant, thanks to three enzymes that contribute to the antioxidant defense system in the body, protecting against further peroxidative damage; thus, it is effective in regulating age-related changes [104]. Among filamentous fungi, many alkaloids and polyketides with antioxidant and neuroprotective properties have been isolated from the polyphyletic group *Deuteromycota*, particularly from *Penicillium* and *Aspergillus* species. In both in vitro and in vivo models, these compounds have demonstrated enhanced free radical scavenging activity, contributing to their neuroprotective effects [105].

Fungal carotenoids, primarily obtained from *Neurospora crassa* and *Fusarium fujikuroi*, especially neurosporaxanthin, have recently attracted attention for their potent antioxidant and neuroprotective activities. In vitro, neurosporaxanthin effectively scavenges reactive oxygen species (ROS) and shields neurons from oxidative stress-induced apoptosis. Notably, a recent animal study confirmed its high bioavailability in mice—surpassing that of β-carotene and β-cryptoxanthin—and revealed that it possesses comparable provitamin A activity [89]. These results highlight its potential as a neuroprotective dietary supplement. However, formulation challenges related to its lipophilic nature remain, pointing to the need for advanced delivery systems that can enhance solubility and central nervous system targeting. While preliminary safety data appear favorable, more comprehensive pharmacokinetic and long-term studies are necessary.

Besides their antioxidant properties, fungal compounds exhibit significant anti-inflammatory effects. Chronic neuroinflammation is characterized by persistent activation of microglia, which leads to excessive production of pro-inflammatory cytokines such as tumor necrosis factor-alpha (TNF-α), interleukin-6 (IL-6), and interleukin-1 beta (IL-1β), which exacerbate neuronal damage [27]. Fungal bioactive compounds have demonstrated important anti-inflammatory properties, modulating neuroinflammatory pathways to reduce neuronal loss [6].

*Antrodia camphorata* (also known as *Taiwanofungus camphoratus*) is a Basidiomycota endemic in Taiwan, growing on Lauraceae family plants, particularly the camphor tree. This fungus, rich in terpenoids, polyphenolics, and polysaccharides, has been used in traditional Chinese medicine for its medicinal properties. One of its major bioactive compounds, antroquinonol, is a tetra-hydro-ubiquinone derivative with various pharmacological activities, particularly in alleviating oxidative stress and inflammation. Antroquinonol has been shown to stimulate the Nrf2 pathway, reducing oxidative stress and Aβ peptide-induced neurodegeneration in AD models. Several studies show that antroquinonol improves learning and memory by decreasing Aβ levels and astrocytosis in the brain [78,79,80]. However, the high cost of production and the absence of human studies limit its development as a neurotherapeutic agent.

In addition to antroquinonol, adenosine isolated from *A. camphorata* has neuritogenic effects through the A2A receptor (A2A-R), which plays a key role in neuroprotection by delaying apoptosis. Adenosine’s ability to cross the blood-brain barrier (BBB) and modulate neuronal and synaptic function positions it as a potential therapeutic agent for stroke-related brain injuries. Furthermore, *A. camphorata*’s compounds may also help mitigate the neurotoxic effects of heme accumulation in intracerebral hemorrhage, with inhibition of heme oxygenase-1 (HO-1), a key enzyme in heme degradation, offering potential protective effects against stroke in animal models [77].

*Ganoderma lucidum* triterpenoids have been shown to suppress the activation of nuclear factor-kappa B (NF-κB), a key regulator of inflammation, thereby reducing cytokine overproduction, like TNF-α and IL-6, and preventing neuroinflammation-associated synaptic dysfunction [68]. Similarly, *Cordyceps sinensis* produces cordycepin, which inhibits microglial activation and reduces oxidative and inflammatory stress in neurodegenerative models [75,106]. Despite these promising findings, clinical validation is still lacking. Moreover, their therapeutic application is constrained by challenges such as low oral bioavailability and variability in the composition of commercial extracts.

*Inonotus obliquus* extracts have also been found to modulate the MAPK and PI3K/Akt signaling pathways, preventing neuroinflammatory responses and promoting neuronal resilience in vitro model [74]. In aging rats, *I. obliquus* extract decreased amyloid precursor protein production and Aβ plaque levels in the hippocampus, along with reduced pro-inflammatory cytokines [73]. However, their pharmacokinetics and in vivo efficacy remain poorly understood, underscoring the need for further research.

Fungal cell membranes are characterized by a high concentration of ergosterol that plays a variety of biological roles, including permeability, fluidity, regulation, and cell cycle control. The average ergosterol can change according to fungal species, culture age, developmental stage (growth phase, hyphae formation, and sporulation), and environmental conditions (pH and temperature), and in *Aspergillus*, *Penicillium*, *Fusarium*, and *Rhizopus* have ranged between 0.4 and 14.3 g/mg dry mass [92].

Ergosterol possesses a wide spectrum of biological properties, as previously reported. It was found to promote neurite outgrowth in in vitro cell models of AD and decreased Aβ accumulation and prolonged lifespan in a *Caenorhabditis elegans* AD transgenic model [87]. Moreover, it is the precursor of vitamin D2 (ergocalciferol), a crucial neuroprotective factor for nerve cells. Vitamin D has been shown to affect biological processes such as cytokine release, cell differentiation, protein expression, signaling, and neurotransmitter release. Furthermore, the use of vitamin D as a supplement has been shown to have a positive effect in several neurodegenerative diseases [88,107].

Other two interesting molecules involved in neuroprotection are monascin and ankaflavin, two yellow pigments produced by *Monoascus purpureus* that have shown anti-inflammatory and antioxidative activity in an AD-mouse model, inhibiting *p*-tau protein expression, Aβ deposition, and ROS formation. They also stimulated the non-amyloidogenic pathway by enhancing sAPPα expression. Therefore, monascin and ankaflavin can be developed as potent neuroprotective agents against Aβ-induced AD, but safety concerns, particularly related to the co-production of mycotoxins like citrinin, raise important questions about their suitability for human use [85].

### 3.2. Modulation of Neurogenic and Neurotransmitter Systems

A fundamental aspect of fungal neuroprotection is their capacity to modulate neurotransmitter systems and promote neurogenesis. Bioactive compounds from *Hericium erinaceus*, such as erinacines and hericenones, have been shown to enhance nerve growth factor (NGF) synthesis, thereby supporting neuronal survival, synaptic plasticity, and regeneration [69]. Preclinical studies indicate that these compounds improve cognitive function, positioning them as promising therapeutic candidates for neurodegenerative diseases characterized by synaptic loss and cognitive impairment. Moreover, a 49-week randomized, placebo-controlled pilot trial (NCT04065061) tested EAHE in 50–75-year-old patients with mild Alzheimer’s disease. Subjects taking three 350 mg capsules of EAHE per day (each capsule contained 5 mg/g erinacine A) showed significant improvements in Mini-Mental State Exam scores versus placebo [72].

Similarly, *Sarcodon* spp. ectomycorrhizal agaricomycetes including *Sarcodon imbricatus*, *S. cyrneus*, and *S. glaucopus* have demonstrated neurogenic potential through their bioactive secondary metabolites [81]. Notable compounds include Cyrneines A and B from *S. cyrneus*, which mimic NGF-mediated neurotrophic activity and induce neurite outgrowth in neuronal cell lines. Additionally, Glaucopine C from *S. glaucopus* enhances neuronal gene expression, while Scabronine A from *S. scabrosus* promotes NGF synthesis in human astrocytoma cells [108]. Further, scabronine derivatives exhibit similar effects on NGF secretion and neurite differentiation in PC12 cells, suggesting that these fungi exert neurogenic properties through pathways involving NF-κB and AP-1 activation [81].

Cyathane diterpenoids, derived from *Cyathus* spp. (family *Nidulariaceae*, phylum *Basidiomycota*), exhibit diverse biological activities, including antibiotic, antifungal, anti-neurodegenerative, antioxidative, and anti-inflammatory effects [82]. Compounds such as striatoids from *C. striatus* have demonstrated significant neurotrophic activity in PC12 cells, while neocyathin from *C. africanus*, along with drimane sesquiterpenoids, promotes neurite outgrowth [83]. These findings underscore their potent NGF-inducing activity and potential as therapeutic agents for neurodegenerative diseases, including AD and PD.

Fungal bioactive compounds also play a critical role in modulating neurotransmitter systems, which are essential for cognitive function, mood regulation, and synaptic plasticity. Neurotransmitter dysregulation, particularly involving dopamine, acetylcholine, and γ-aminobutyric acid (GABA), is a hallmark of neurodegenerative diseases such as AD and PD [35]. Studies have shown that *Hericium erinaceus* compounds enhance cholinergic signaling by upregulating acetylcholine levels and inhibiting acetylcholinesterase (AChE), leading to improved memory and learning abilities. Additionally, erinacines from *H. erinaceus* stimulate NGF synthesis, promoting synaptic regeneration and neuroplasticity [70].

Polysaccharides from *Ganoderma lucidum* have been found to regulate dopamine metabolism, preventing dopaminergic neuron depletion in PD models [68]. Cordycepin from *Cordyceps sinensis* has also been reported to modulate serotonin and glutamate receptors, suggesting antidepressant and neuroprotective effects that could benefit individuals with neurodegenerative diseases and age-related cognitive decline [76].

Furthermore, fungal-derived GABA has been identified as a crucial modulator of neuronal excitability, potentially mitigating excitotoxic damage seen in conditions such as Huntington’s disease and amyotrophic lateral sclerosis [92]. Filamentous fungi, particularly *Monascus purpureus* and *Rhizopus oligosporus*, produce high levels of GABA in response to environmental acid stress [92]. GABA plays a pivotal role in inhibitory neurotransmission across central and peripheral pathways, and its dysregulation has been implicated in multiple neurodegenerative and neuropsychiatric disorders, including HD, PD, senile dementia, seizures, AD, stiff person syndrome, and schizophrenia [109].

Overall, these fungal-derived bioactive compounds hold significant therapeutic promise in modulating neurogenic pathways and neurotransmitter systems, offering potential interventions for neurodegenerative diseases and cognitive decline.

### 3.3. Modulation of Mitochondrial Dynamics

Mitochondria are essential organelles responsible for energy production, calcium regulation, and apoptosis. Mitochondrial dysfunction is implicated in various neurodegenerative diseases, leading to neuronal damage and cell death. Mitochondrial dysfunction, often related to oxidative stress, contributes to the progression of neurological disorders. Interestingly, fungal-derived compounds have shown promise in restoring mitochondrial health. Extracts from *Inonotus obliquus* have been shown to enhance mitochondrial membrane potential and improve cellular bioenergetics, thereby protecting neurons from apoptosis and functional decline [73,74]. In particular, a triterpenoid named 2α-hydroxy-inotodiol recently isolated from *I. obliquus* demonstrated remarkable neuroprotective activity mediated by Nrf2 and BDNF/TrkB/ERK/CREB pathways, and further ameliorated mitochondrial damage induced by ROS [73].

Bioactive compounds from *Cordyceps* spp. have been shown to alleviate mitochondrial dysfunction, to reduce apoptosis, and to improve levels of antioxidant enzymes, contributing to neuroprotection [106]. Particularly, extracts from *C. synensis* were shown to improve the activities of the mitochondrial complexes I-IV and ATP production, to restore the mitochondrial membrane potential, and to inhibit the release of mitochondrial apoptogenic factors [75,110].

Ganoderic acids, a family of triterpenoids isolated from *Ganoderma lucidum*, increased the levels of SOD to inhibit the production of ROS, thereby preserving the integrity of the mitochondrial membranes and improving the mitochondrial membrane potential of hippocampal neurons [3,111]. Additionally, *G. lucidum* polysaccharides modulated the activation of both the AMPK/mTOR and PINK1/Parkin signaling pathways regulating the stress resistance, mitochondrial maintenance, and autophagy response in a Parkinson’s mouse model [68]. Other terpenoids have also been shown to regulate autophagy, a crucial process in removing damaged mitochondria, which helps maintain neuronal homeostasis and prevents neurodegenerative progression [112]. These findings suggest that fungal bioactive compounds play a significant role in maintaining mitochondrial health, thereby offering potential therapeutic strategies for neurodegenerative diseases.

## 4. Conclusions

Fungal-derived bioactive compounds represent a rapidly emerging field in anti-aging research, offering novel therapeutic potential through their diverse biochemical properties. Here, we have reviewed some biologically active fungal metabolites, such as polysaccharides, terpenoids, and phenolic compounds that have demonstrated significant antioxidant, anti-inflammatory, and neuroprotective activities in both in vitro and in vivo studies. Notably, many compounds, such as ergothioneine, cordycepin, and β-glucans, have been shown to regulate cellular oxidative stress, enhance mitochondrial function, and modulate both neurogenic and neurotransmitter systems, all critical factors in aging and neurodegenerative diseases.

Overall, a major limitation is the significant gap between preclinical findings and clinical translation. Despite promising findings, several challenges must be addressed before these compounds can be translated into clinical applications. While in vitro and in vivo data support the therapeutic potential of fungal metabolites, comprehensive and deeper preclinical and clinical trials addressing their efficacy in aging and neurodegeneration are lacking, limiting our ability to determine their pharmacokinetics, bioavailability, and long-term effects in humans. Moreover, variability in fungal strain composition, inconsistencies in extraction protocols, the lack of standardized bioactive content, and limited pharmacokinetic data contribute to inconsistencies in results.

Future research should prioritize studies to better elucidate the molecular interactions of these bioactive compounds with pathways involved in neurodegeneration and in neuroprotection, together with advancements in biotechnological production methods to enhance yield and purity. With continued interdisciplinary efforts, fungal bioactive compounds may pave the way for innovative interventions on neurodegenerative disease, contributing to healthier aging and longevity.

## Figures and Tables

**Table 2:** Summary of main fungal compounds with proven biological function.

Fungal Source	Main Molecules Extracted	Extraction Method	Effects and Bioactivities	Concentrations	Experimental Validation	References
*Ganoderma lucidum* (Curtis) P.Karst. known as Reishi	Ganoderic acid A; β-glucans	Ethanol extraction (basidiome); hot-water extraction (polysaccharides)	- Inhibites of NF-κB and MAPK signaling; - Activates of autophagy-related pathways (LC3B, Beclin-1, Axl); - Free radicals scavenging (O_2_^−^, OH^·^)	Ganoderic acid A: IC_50_ ≈ 25–35 μM; β-glucans EC_50_ ≈ 150 μg/mL	In vitro: HT22 BV2 In vivo: MPTP-lesioned mice, Aβ-injected C57BL/6 J mice	[66,67,68]
*Hericium erinaceus* (Bull.) Person, known as Lion’s Mane	Hericenones E; Erinacine A	Methanol extraction (basidiome); ethanol extraction (mycelium)	- Regulates NGF synthesis via MEK/ERK and PI3K-Akt; - Upregulates BDNF and neurogenesis markers in hippocampal neurons	Hericenone E: active ≥ 10 μM; Erinacine A: EC_50_ ≈ 2.0 μg/mL	In vitro: PC12 cell line In vivo: rat model 3-AP–induced cerebellar ataxia; APP/PS1 mice Clinical: Mild AD trial with EAHE (NCT04065061)	[69,70,71,72]
*Inonotus obliquus* (Fr.) Pilat, known as Chaga	3,4-Dihydroxybenzalacetone; polysaccharides	Ethyl acetate extraction; hot-water extraction	- Activates Nrf2/ARE pathway; - Increases HO-1, SOD1, GPx; - Stabilizes mitochondria	3,4-Dihydroxybenzalacetone: IC_50_ ≈ 12.5 μM	In vitro: PC12; SH-SY5Y cell lines In vivo: Aβ-injected rats	[73,74]
*Cordyceps sinensis* (Bertk.) Sacc. known as Zombie Fungus	Cordycepin	Ethanol or Methanol extraction; silica-gel chromatography	- Triggers microglia polarization through OXPHOS and glycolysis pathways; - Modulates AMPK/mTOR signaling; - Inhibits TLR4/NF-κB axis; - Stabilizes mitochondrial dynamics via Drp1/Mfn1	Cordycepin: IC_50_ ≈ 20–30 μM	In vitro: BV2 and SH-SY5Y; PC12 In vivo: APP/PS1 mice, MPTP-lesioned mice	[75,76]
*Antrodia camphorate* (Chang and Chou)	Antroquinonol	Ethanol extraction; HPLC purification	- Inhibits Aβ aggregation and GSK-3β and BACE1 expression; - Activates PI3K/Akt; - Inhibits NLRP3 inflammasome; - Activates Nrf2 pathway	Antroquinonol: IC_50_ ≈ 1.2 μM	In vitro: PC12 cell line In vivo: 3xTg mice; APP mice	[77,78,79,80]
*Sarcodon* spp.	Cyrneines A	Acetone/methanol extraction; silica-gel chromatography	- Mimics NGF via TrkA; - Activates Ras/MAPK and PI3K/Akt; - Promotes neuritogenesis	Cyrneine A: EC_50_ ≈ 1.0 μM	In vitro: PC12 cell line	[81]
*Cyathus* spp.	Striatoids	Chloroform/methanol extraction; silica-gel purification	- Activates NGF- neurite outgrowth; - Anti-inflammatory activities modulating iNOS and COX-2 proteins	Striatoid EC_50_ ≈ 2.5–5 μM	In vitro: PC12 cell line; BV2 cells	[82,83]
*Monascus* spp.	Monascin; Ankaflavin	Solid-state fermentation on rice; ethyl acetate extraction	- Inhibits oxidative stress; - Induces MAPK activation; - Inhibits BACE and apo E expression; - Downregulates iNOS and COX-2 expression	Monascin: IC_50_ ≈ 25 μM; Ankaflavin: IC_50_ value of 15 microg/mL	In vitro: PC-12 cells In vivo: Sprague Dawley rats	[84,85]
Various Fungi	Ergosterol	Lipid extraction (chloroform:methanol); crystallization	- Activates ROS scavenging-Inhibits NADPH oxidase activity; - Promotes Nrf2/SOD-1 and RICTOR/Akt/GSK-3β signaling pathways; - Inhibits ionotropic glutamate receptor overexpression via EGR-1 regulation; - Induces neurite outgrowth via Ten-4/ERK/CREB/GAP-43 pathway and decreases Aβ accumulation	Ergosterol: active ≥ 20 μM	In vitro: BV2 cell line; Neuro2a cell line In vivo: *Caenorhabditis elegans*	[86,87,88]
*Neurospora crassa* (Shear & Dodge) and Fusarium fujikuroi (J. Shld.)	Neurosporaxanthin; β-carotene	Solvent extraction (acetone, ethanol); mechanical cell disruption; HPLC purification	- Activates RARs and RXRs	EC_50_ ≈ 5–10 μM	In vivo: C57BL/6 wild-type	[89]
